# First person – Sarah K. Lamar

**DOI:** 10.1242/bio.059648

**Published:** 2022-10-17

## Abstract

First Person is a series of interviews with the first authors of a selection of papers published in Biology Open, helping researchers promote themselves alongside their papers. Sarah K. Lamar is first author on ‘ Investigating the link between morphological characteristics and diet in an island population of omnivorous reptiles (*Sphenodon punctatus*)’, published in BiO. Sarah is a PhD candidate and personal research assistant to the Head of the School of Biological Sciences in the lab of Professor Nicola Nelson and Dr. Diane Ormsby (co-chairs) at Victoria University of Wellington. Her research interests rest at the intersection of herpetology, evolution, statistics, and conservation.


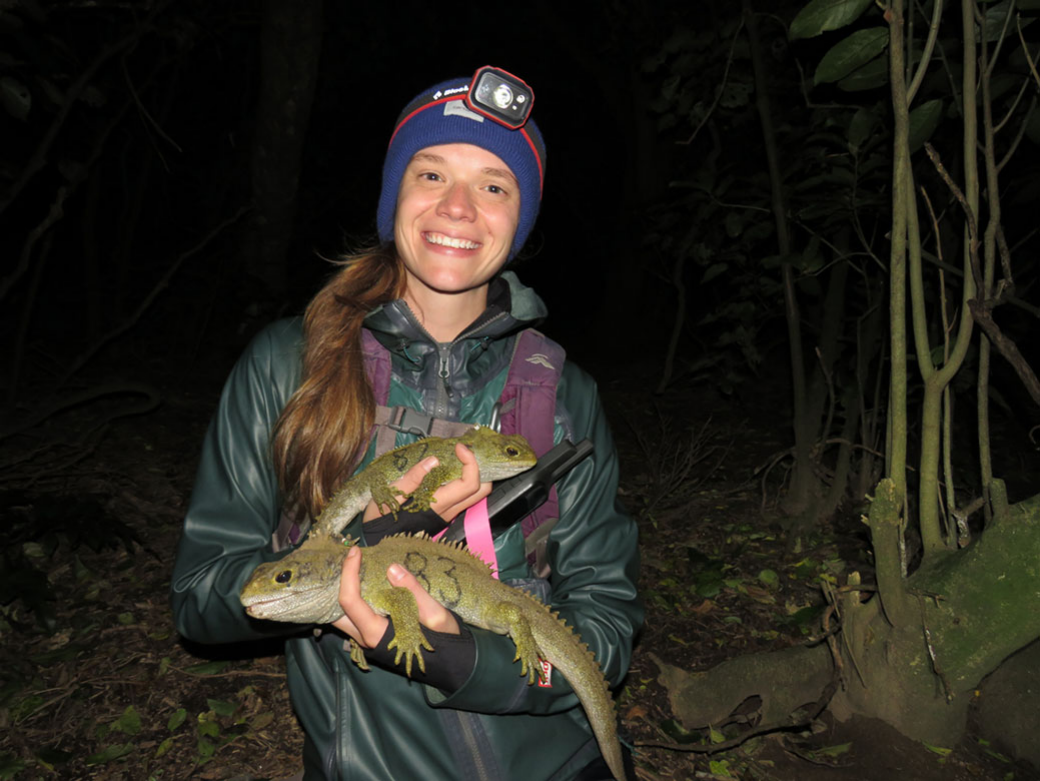


**Sarah K. Lamar**

**Describe your scientific journey and your current research focus**

My journey has not been a straight line – I have a background in invasion ecology, veterinary nursing, and amphibian monitoring. I took some time off school to figure things out and work full time, and it was the best decision I could ever have made. My first postgraduate mentor, Dr. Charlyn Partridge, deserves huge credit for inspiring me to follow my creativity in science.

My current research focuses, broadly, on fitness in male tuatara. More generally, I'm interested in asking creative questions about evolutionary and physiological challenges to herpetofauna conservation.

“I took some time off school to figure things out and work full time, and it was the best decision I could ever have made.”

**Who or what inspired you to become a scientist?**

I grew up in the southern USA, and my early years were filled with catching bugs, turtles, and snakes (much to my parents’ chagrin). Instead of deciding to abandon these interests as I got older, I pivoted into asking deeper questions about them.

**How would you explain the main finding of your paper?**

Our research asks questions about the disparity in food items available to tuatara that have been reintroduced to restored habitat on Aotearoa New Zealand's mainland from offshore islands. We found that a significant percentage of the dietary carbon consumed by tuatara living offshore had a marine (seabird) signature – meaning tuatara, particularly large ones, eat a lot of seabirds.

**What are the potential implications of this finding for your field of research?**

Tuatara, and other native *taonga* species in Oceania, are being reintroduced to restored mainland habitat. However, the mainland is now largely free from seabird colonies – meaning the dietary opportunity for seabird intake is either absent or much reduced. Seabirds provide a boon of nutrients available to tuatara from nowhere else – and this raises serious questions about the physiological implications translocated populations of tuatara may be facing.

**Figure:**
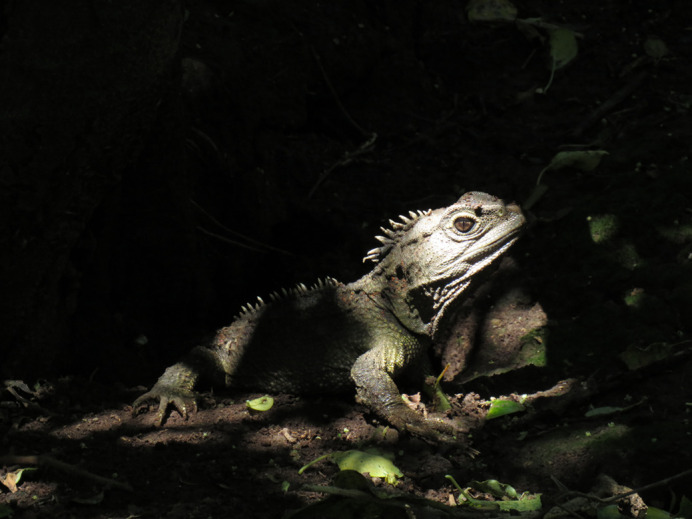
A male tuatara (*Sphenodon punctatus*) basks in a sunbeam managing to break through the forest canopy on Takapourewa/Stephens Island, New Zealand.

**Which part of this research project was the most rewarding?**

Doing science that's rooted in interesting physiological theory (in this case, functional morphology) but has immediate, practical implications for the conservation of threatened species is immensely rewarding and makes a real difference in the world.

“Doing science that's rooted in interesting physiological theory … but has immediate, practical implications for the conservation of threatened species is immensely rewarding…”

**What do you enjoy most about being an early-career researcher?**

I love the relative freedom of opportunity during the early-career stage of research – I've met a lot of great collaborators and taken part in interesting ‘side’ projects because of the freedom in my schedule – tuatara viruses, Leiopelmatid habitat studies, and microbiome work!

**What piece of advice would you give to the next generation of researchers?**

Find something you care about and stick with it.

**What's next for you?**

I'm still figuring that out! I love research and conservation, and if this sounds like something related to an opportunity you might know of, feel free to reach out at sarah.lamar@vuw.ac.nz
